# Highly Active Nickel (II) Oxide-Supported Cerium Oxide Catalysts for Valorization of Glycerol into Oxygenated Fuel Additives

**DOI:** 10.3390/ma16134713

**Published:** 2023-06-29

**Authors:** Jimmy Nelson Appaturi, Pedro Maireles-Torres, Taghrid S. Alomar, Najla AlMasoud, Zeinhom M. El-Bahy, Tau Chuan Ling, Eng-Poh Ng

**Affiliations:** 1 School of Biological Sciences, Universiti Sains Malaysia, Gelugor 11800, Penang, Malaysia; 2 Departamento de Química Inorgánica Cristalografía y Mineralogía (Unidad Asociada al ICP-CSIC), Facultad de Ciencias Campus de Teatinos, Universidad de Málaga, 29071 Málaga, Spain; maireles@uma.es; 3 Department of Chemistry, College of Science, Princess Nourah bint Abdulrahman University, P.O. Box 84428, Riyadh 11671, Saudi Arabia; tsalomar@pnu.edu.sa (T.S.A.); nsalmasoud@pnu.edu.sa (N.A.); 4 Department of Chemistry, Faculty of Science, Al-Azhar University, Nasr City, Cairo 11884, Egypt; zeinelbahy@azhar.edu.eg; 5 Institute of Biological Sciences, Faculty of Science, University of Malaya, Kuala Lumpur 50603, Malaysia; tcling@um.edu.my; 6 School of Chemical Sciences, Universiti Sains Malaysia, Gelugor 11800, Penang, Malaysia

**Keywords:** glycerol acetylation, sol-gel technique, NiO-incorporated CeO_2_, triacetin, automotive fuels

## Abstract

Acetylation of glycerol to yield monoacetin (MAT), diacetin (DAT), and triacetin (TAT) over NiO-supported CeO_2_ (*x*NiO/CeO_2_) catalysts is reported. The catalysts were synthesized utilizing a sol-gel technique, whereby different quantities of NiO (*x* = 9, 27, and 45 wt%) were supported onto the CeO_2_ substrate, and hexadecyltrimethylammonium bromide (CTABr) served as a porogen. The utilization of EDX elemental mapping analysis confirmed the existence of evenly distributed Ni^2+^ ion and octahedral NiO nanoparticles on the CeO_2_ surface through the DRS UV-Vis spectroscopy. The most active catalyst is 27NiO/CeO_2_ based on TAT selectivity in the glycerol acetylation with ethanoic acid, attaining 97.6% glycerol conversion with 70.5% selectivity to TAT at 170 °C with a 1:10 glycerol/ethanoic acid molar ratio for 30 min using a non-microwave instant heating reactor. The 27NiO/CeO_2_ is reusable without significant decline in catalytic performance after ten consecutive reaction cycles, indicating high structure stability with accessible active acidity.

## 1. Introduction

One of the most significant contributions in reducing greenhouse gas emissions is the production of biodiesel via the transesterification of vegetable oil or animal fat [1,2,3,4]. Meanwhile, glycerol is a by-product of biodiesel production that accounts for about 10% of the total production [5,6]. Due to the surplus of glycerol and the need to keep the chemical industry competitive, current research is mainly focused on the catalytic methods for converting glycerol into value-added compounds [1]. So far, various approaches to valorize glycerol, such as acetylation [7], aromatization [8], dehydration [9], esterification [10], hydrogenolysis [11], etherification [12], and acetalization [13,14], have been developed via the heterogeneous catalysis route.

Acetylation of glycerol has recently piqued researchers’ interest due to the industrial significance of the end products. Mono-, di-, and triacetins (MATs, DATs, and TATs) are the three main products of the process. These products show broad and promising applications in many industries. Specifically, MAT is used in manufacturing explosives (dynamite), tanning animal skin for leather, and as a solvent for dyes [15], and DAT is used as a plasticizer, a solvent for dyes, and a softening agent [16,17]. TAT is the most difficult synthesized acetin produced in the final phase. It has been used as a high-value oxygenated fuel/diesel additive to promote clean combustion and as an anti-occlusive agent since it raises the octane number [17,18]. Furthermore, TAT can serve as an anti-knocking agent for gasoline. Adding TAT into biodiesel also improves cold flow properties and viscosity, and decreases the cloud and pour points of biodiesel [15,19].

Mineral acids, such as HCl, H_2_SO_4_ and HNO_3_, have traditionally been utilized as homogenous catalysts in the acetylation of glycerol [20]. However, these acids have numerous drawbacks, including hazardous operating conditions, equipment corrosion problems, tedious catalyst separation, and low catalyst reusability [15]. Numerous attempts have been made to enhance the ecological sustainability of this procedure through the utilization of eco-friendly heterogeneous solid catalysts free of solvents in catalytic operations. Various catalysts have been studied, such as CeO_2_–Al_2_O_3_ [21], H_3_PW_12_O_40_/silica [22], H_3_PW_12_O_40_/carbon [23], and HZSM-5 [24]. Although certain catalysts have exhibited potential catalytic activity, attaining high reaction conversion and desired TAT selectivity have proven to be challenging in most cases. Moreover, some catalytic systems need long reaction durations (2 to 6 h) [25,26], as well as an excessive amount of acetylating agents [27]. Hence, it is imperative to enhance the efficiency of the catalytic acetylation reaction for urgent industrial needs.

In addition to the utilization of mineral acids in the process of acetylation, the incorporation of metal oxide, specifically NiO, has been identified as a viable approach to enhance the reaction activity. The cubic lattice structure of NiO renders it a crucial metal oxide in catalysis. Recently, there has been a growing interest among researchers in exploring Ni-based catalysts owing to their substantial availability and high catalytic activity [28]. NiO is an example of a p-type semiconductor and it is able to adsorb various oxygen species on its surface in moderate environments. However, NiO is easily reduced to Ni^0^, making a single NiO as a dependable catalyst with improved catalytic properties a difficult task [29]. Thus, composite oxide with relatively higher stability is needed to circumvent this problem.

The utilization of cerium (IV) oxide (CeO_2_) provides good support thanks to its distinct chemical and physical properties. The presence of ceria in both Ce^3+^ and Ce^4+^ valence states allows for a reversible valence state change, resulting in favorable redox properties [30]. Additionally, this leads to an abundance of structural defects, specifically oxygen vacancies, which can enhance the lattice oxygen mobility, active oxygen levels, and oxygen storage capacity during the reaction process [30,31]. Ceria has been demonstrated to be an oxide support that readily interacts with active metal phases. Therefore, the incorporation of CeO_2_ support in nickel-based catalysts would result in enhanced metal–support interaction, reduced particle agglomeration, and improved metallic dispersion [32].

This study describes the development of a highly effective catalyst, *x*NiO/CeO_2_, comprised of NiO supported on CeO_2_ particles for the acetylation of glycerol in the production of triacetin (TAT). To achieve this ultimate goal, the catalysts were first designed and synthesized by incorporating different amounts of Ni (*x* = 9, 27, and 45 wt.%) via a facile sol-gel treatment technique. The catalytic activity and selectivity for a given reaction can be influenced by the selection of varying weight percentages of NiO and CeO_2_. Hence, the objective was to determine the optimum NiO:CeO_2_ composition ratio that exhibits cooperative interaction between the two components, which gives the greatest activity and selectivity for the synthesis of TAT. The study then focused on the influence of reaction parameters (e.g., reaction temperature and time, glycerol to ethanoic acid ratio, and catalyst dosage) on the performance of catalytic reaction. We believe that the current study greatly benefits the manufacture of TAT biodiesel by designing and developing an active and selective *x*NiO/CeO_2_ catalyst.

## 2. Experimental

### 2.1. Catalyst Preparation

**Scheme 1:**
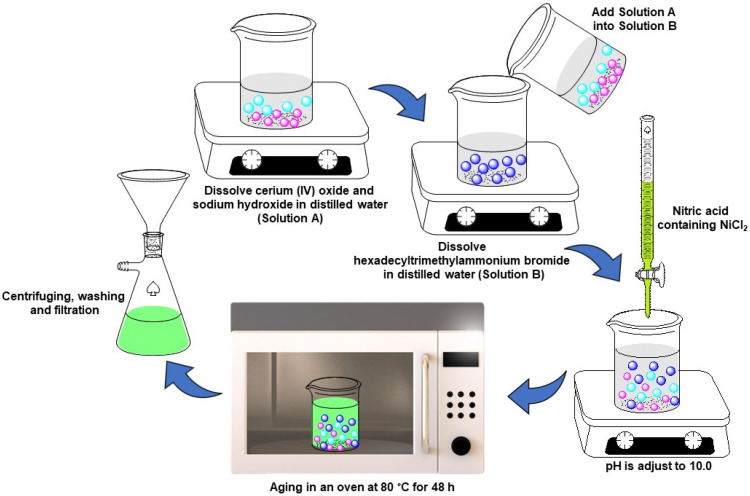
Synthesis route of *x*NiO/CeO_2_ catalysts.

The synthesis of *x*NiO/CeO_2_ catalysts (*x* = 9, 27, and 45 wt.% of NiO) was carried out using the sol-gel technique according to the modified method of Andas et al. [33]. The typical synthesis process of the *x*NiO/CeO_2_ catalysts is schematically illustrated in Scheme 1. For the preparation of the 45NiO/CeO_2_ catalyst, a mixture of 50.00 g of distilled water, 1.50 g of cerium (IV) oxide (Sigma Aldrich, 99.9%, St. Louis, MO, USA), and 20.00 g of NaOH (Sigma Aldrich, 98%, St. Louis, MO, USA) was first mixed (400 rpm) for 20 min. This solution was then added to another clear solution containing 2.85 g of hexadecyltrimethylammonium bromide (Sigma Aldrich, 99%, St. Louis, MO, USA) and 25.00 g of distilled water. The final solution was further stirred (400 rpm) for another 17 h at 80 °C. Next, the pH of the mixture was adjusted to 10.0 using an HNO_3_ solution (39.00 g, 3 M) that contained 1.49 g of NiCl_2_ (Merck, 98%, Rahway, NJ, USA). At this pH, hexadecyltrimethylammonium bromide molecules tend to self-assemble into micelles, creating an ideal environment for the subsequent integration of metal precursors for catalyst production with mesoporosity [34]. The mixture was kept agitated for 2 h prior to aging in an oven at 80 °C for 48 h. The resulting precipitate was separated via centrifugation (7000 rpm, 5 min), washed until pH 7, and dried (90 °C, 10 h) before being calcined (580 °C, 5 h) to eliminate the organic porogen template. A similar protocol was used to prepare other *x*NiO/CeO_2_ catalysts via varying the NiO amount where *x* = 9 (0.30 g NiCl_2_) and *x* = 27 (0.90 g NiCl_2_).

### 2.2. Catalyst Characterization

The X-ray diffractograms (XRDs) were obtained using a PANalytical X’Pert Pro diffractometer, which employed CuKα radiation with a wavelength of 1.5418 Å, a voltage of 40 kV, and a current of 10 mA. The scanning range was from 2θ = 20° to 65°, with a scanning rate of 0.2° per min and a step size of 0.02°. The surface area and pore size distribution of catalysts were characterized using the N_2_ adsorption–desorption isothermal analysis obtained at −196 °C through a Micromeritics ASAP 2010 analyzer. The catalyst powder (0.0900 mg) was first degassed for 12 h at 300 °C and 10^−4^ Pa in the degassing port of the analyzer. The surface area was computed using the Brunauer–Emmett–Teller (BET) method, while the pore size distribution was calculated using the Barrett, Joyner, and Halenda (BJH) method. The diffuse reflectance (DRS) UV-Vis spectra of catalysts were obtained on a Perkin Elmer Lamda 35 spectrometer. The field emission scanning electron microscopy (FESEM) and energy dispersive X-ray (EDS) analyses for morphological and elemental mapping investigations were performed on FEI’s Quanta FEG 650 microscope equipped with an Oxford XMax 50 Silicon Drift EDS detector. The transmission electron microscopy (TEM) study was performed on a Philips CM-12 TEM microscope. The samples in methanol solvent were first sonicated for 10 min before being deposited on copper grids for imaging analysis. The acidity of catalysts was characterized by the ammonia temperature programmed desorption (TPD-NH_3_) technique using a Thermo Electron TPDRO 1100. Prior to analysis, the sample (0.060 g) was degassed at 500 °C overnight, followed by NH_3_ adsorption for 30 min. The ammonia probe molecule was then desorbed from the sample surface from 50 to 1000 °C at a heating rate of 10 °C min^−1^. The thermogravimetric analysis (TGA) was performed on Mettler Toledo 851e equipment. The temperature was measured from 50 to 900 °C at a heating rate of 20 °C min^−1^ and air flow rate of 40 mL min^−1^. The amounts of cerium and nickel in the samples were measured using a Perkin Elmer Optima 4300DV ICP-OES spectrometer. Before measurement, the sample powder was first dissolved in an aqua regia (HNO_3_:HCl = 1:3) and subjected to microwave digestion at 200 °C for 20 min (800 W) using a CEM MARS 6 microwave digester.

### 2.3. Catalytic Reaction Study and Products Analysis

The process of acetylating glycerol was conducted within a temperature range of 130–170 °C, utilizing a non-microwave instant heating reactor (Anton Paar’s Monowave 50). Initially, 0.040 g of *x*NiO/CeO_2_ catalyst was first activated at 250 °C for 1 h before it was added with 0.139 g of glycerol (QReC, 99.5%) and 0.900 g of ethanoic acid (Sigma-Aldrich, St. Louis, MO, USA) into a glass tube (10 cm^3^). The mixture was sealed with a silicone cap and rapidly heated to a desired temperature (150–170 °C) and kept at a plateau for 5–60 min. After the reaction, the tube together with the mixture were subjected to high-speed centrifugation (10,000 rpm, 10 min) to separate the solid catalyst. The remaining liquid product was then analyzed using an Agilent’s HP 6890 GC instrument with an FID detector. The glycerol conversion and products’ selectivity were determined using the following equations (toluene as internal standard):

(1) $$Glycerol\;conversion\left(\%\right)=\left(1-\frac{{mol}_{glycerol,t}}{{mol}_{glycerol,0}}\right)\times 100$$

(2) $$Selectivity\left(\%\right)=\frac{{mol}_{MAT}\;or\;{mol}_{DAT}\;or\;{mol}_{TAT}}{{mol}_{Glycerol}\;converted}\times 100$$

where mol_glycerol,0_ and mol_glycerol,t_ are the amount of glycerol in mol before and after acetylation reaction at t min. Meanwhile, mol_MAT_, mol_DAT_, mol_TAT_, and mol_Glycerol_ are the amounts of MAT, DAT, TAT, and glycerol in mol, respectively. All the quantitative analyses were performed using the absolute calibration method where the known amounts of reactant (glycerol) and products (MAT, DAT, and TAT) are used for plotting of calibration curves. The recovered catalyst was soaked and washed three times with diethyl ether (30 mL), and air dried before being activated once more (250 °C, 1 h) for the next cycles of reaction.

## 3. Results and Discussion

### 3.1. Characterizations of xNiO/CeO_2_ Catalysts

**Figure 1:**
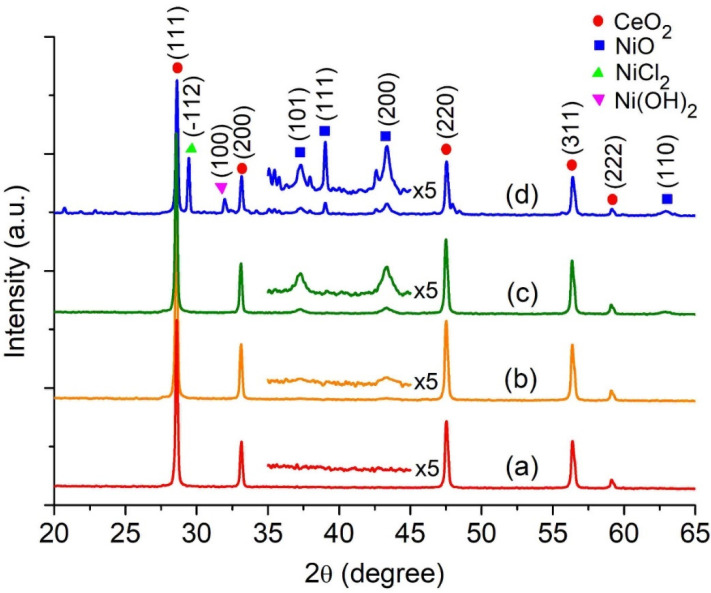
XRD patterns of (**a**) CeO_2_, (**b**) 9NiO/CeO_2_, (**c**) 27NiO/CeO_2_, and (**d**) 45NiO/CeO_2_.

NiO/CeO_2_ catalysts were first studied with the XRD technique, and the wide-angle XRD pattern is shown in Figure 1. As seen, all prepared solids show five major peaks at 2θ angles of 28.5°, 33.0°, 47.5°, 56.3°, and 59.2° attributed to the (111), (200), (220), (311), and (222) planes of CeO_2_, respectively [35]. The crystallography data also correspond to the cubic fluorite structure (Fm3m) of CeO_2_ (JCPDF No. 43-1002). Additionally, several weak diffraction peaks due to (101), (111), (200), and (110) crystal planes of NiO are also detected at 2θ = 37.2°, 38.9°, 43.3°, and 62.9° by 9NiO/CeO_2_, 27NiO/CeO_2_, and 45NiO/CeO_2_. These peaks become more intense with increasing the NiO content. The presence of these peaks can be explained by the presence of NiO particles deposited on the sample surfaces compared to that of pristine CeO_2_. However, the XRD peak intensity of NiO is weak despite the high Ni content in the catalyst. This phenomenon could be explained by the very small crystallite size of NiO, which is distributed evenly on the CeO_2_ support. As a result, it causes the peaks to spread and lose intensity, lowering the diffraction intensity. Moreover, a few impurities peaks due to NiCl_2_ and Ni(OH)_2_ are also found in the 45NiO/CeO_2_ catalyst (at 2θ = 29.5°, 31.9°), where the results are well corroborated with the JCPDF No. 002-0765 [36] and JCPDS No. 14-0117, respectively [37]. However, these peaks are not observed in 9NiO/CeO_2_ and 27NiO/CeO_2_ catalysts with less NiO content, suggesting only pure NiO and CeO_2_ crystal phases are obtained in both solids.

**Figure 2:**
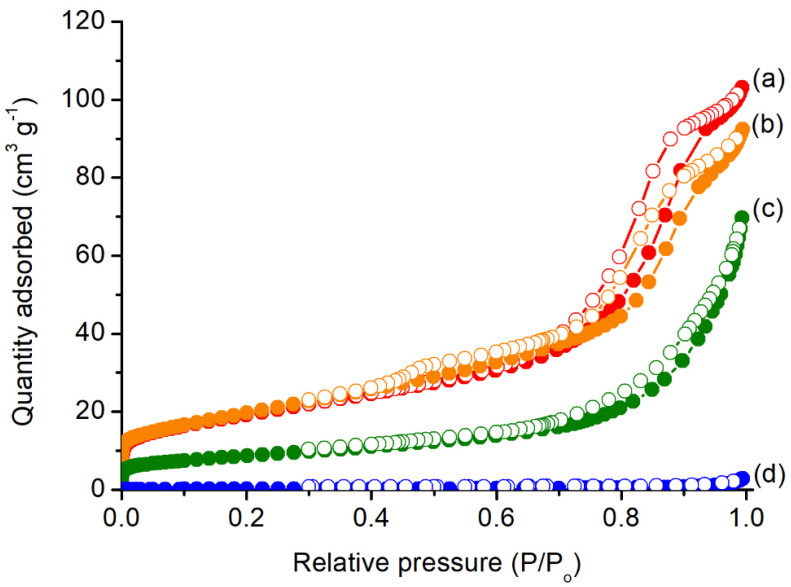
Nitrogen adsorption–desorption isotherms of (**a**) CeO_2_, (**b**) 9NiO/CeO_2_, (**c**) 27NiO/CeO_2_, and (**d**) 45NiO/CeO_2_.

Figure 2 shows the nitrogen adsorption–desorption isotherms for the *x*NiO/CeO_2_ solids. The CeO_2_ initially has a specific surface area (S_BET_) and a total pore volume (V_Total_) of 69 m^2^/g and 0.15 cm^3^/g, respectively (Table 1). The solid material demonstrates a type IV adsorption isotherm and H1 hysteresis loop, as per the IUPAC classification, indicating the presence of mesoporous structure having a small pore mouth and wide body (ink-bottle pores) with an average pore size of 9.86 nm (Figure 2a). The closure point of the hysteresis loop at P/P_o_ = 0.4 shows that adding CTABr to the synthesis of *x*NiO/CeO_2_ results in the generation of an extra new mesopore system (Figure 2b). Yet, when the nickel level increases, the fundamental mesostructure identity of the solid gradually diminishes. For instance, the 9NiO/CeO_2_ retains its type IV isotherm and H3 hysteresis loop features but has lower nitrogen uptake. As a result, the average pore size and total pore volume are slightly decreased. On the other hand, the specific surface area remains almost intact due to the generation of secondary mesopores by the CTABr mesoporogen. Upon further increasing the nickel content, the porosity gradually decreases (low surface area, micropore surface area, and total pore volume), leading to narrower average pore sizes. In addition, the initial ink-bottle pore shape also changes to a slit shape (for 27NiO/CeO_2_) and finally the inner pores of the solid are fully covered (for 45NiO/CeO_2_). Such a phenomenon is associated with partial or complete pore blockage by the NiO nanoparticles [38].

**Figure 3:**
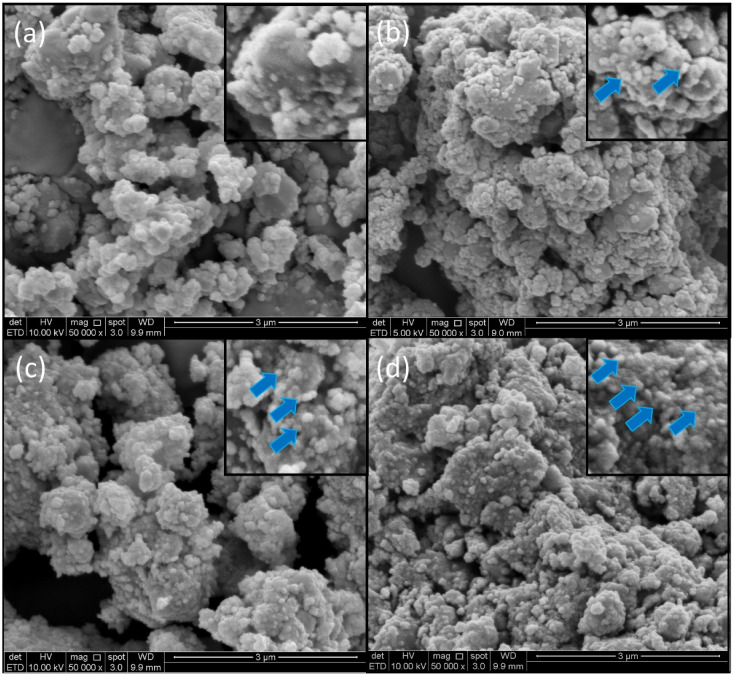
SEM images of (**a**) CeO_2_, (**b**) 9NiO/CeO_2_, (**c**) 27NiO/CeO_2_, and (**d**) 45NiO/CeO_2_. The blue arrows show the NiO nanoparticles deposited on the CeO_2_ support.

**Figure 4:**
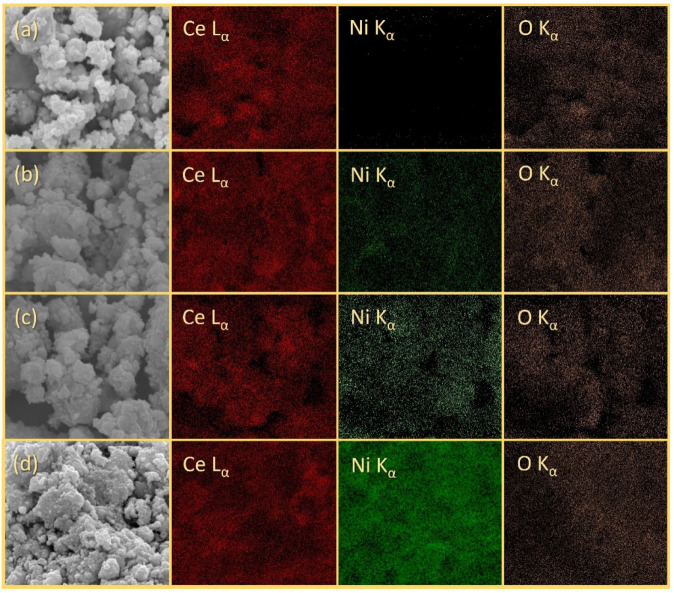
Elemental distribution mapping images of (**a**) CeO_2_, (**b**) 9NiO/CeO_2_, (**c**) 27NiO/CeO_2_, and (**d**) 45NiO/CeO_2_.

SEM analysis is performed to investigate the morphological features of samples after sol-gel treatment. As seen, the CeO_2_ consists of agglomerated irregular-shaped particles with a size of 125 ± 23 nm (Figure 3a). Upon impregnating with various amounts of NiO, the density of bright nanoparticles also increases and they are uniformly distributed all over the surface of the samples (Figure 3b–d). The identity of bright spots was further investigated using EDX elemental mapping analysis where Ce, Ni, and O elements were mapped onto the SEM images (Figure 4). For the pristine CeO_2_ sample, only Ce and O elements are detected due to the CeO_2_ support itself. For *x*NiO/CeO_2_ solids, the micro-analysis reveals that the Ni element is enriched on the solid surface relative to the amount of NiO introduced. In addition, the Ni element is also well-scattered on the solid surface without segregation, further proving the successful functionalization process.

**Figure 5:**
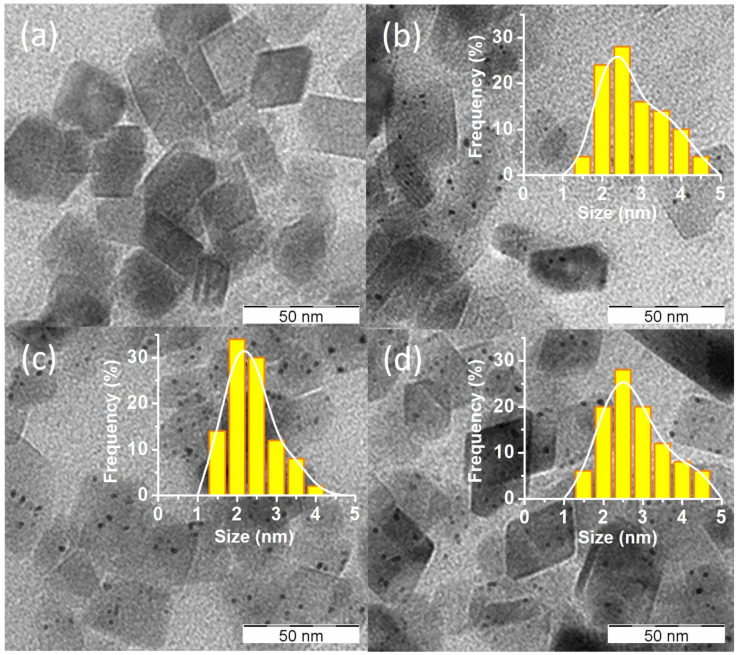
TEM images of (**a**) CeO_2_, (**b**) 9NiO/CeO_2_, (**c**) 27NiO/CeO_2_, and (**d**) 45NiO/CeO_2_ where the presence of NiO nanoparticles (dark spots) and their size distribution (inset) on the CeO_2_ support are shown.

The microscopic visualization of NiO nanoparticles was further carried out using the TEM microscopy technique. Similar to the SEM/EDX study, uniform dispersion of NiO nanoparticles (approx. 2–3 nm) on the surface of CeO_2_ solid is seen in the *x*NiO/CeO_2_ samples (as dark spots) (Figure 5). On the other hand, the morphology and size of CeO_2_ crystals (ca. 165 nm in grain shape) is unaffected upon sol-gel and calcination treatments. As a result, the microscopy results are in accordance with the XRD findings, viz. no substantial NiO particles are dispersed on the CeO_2_ surface.

**Figure 6:**
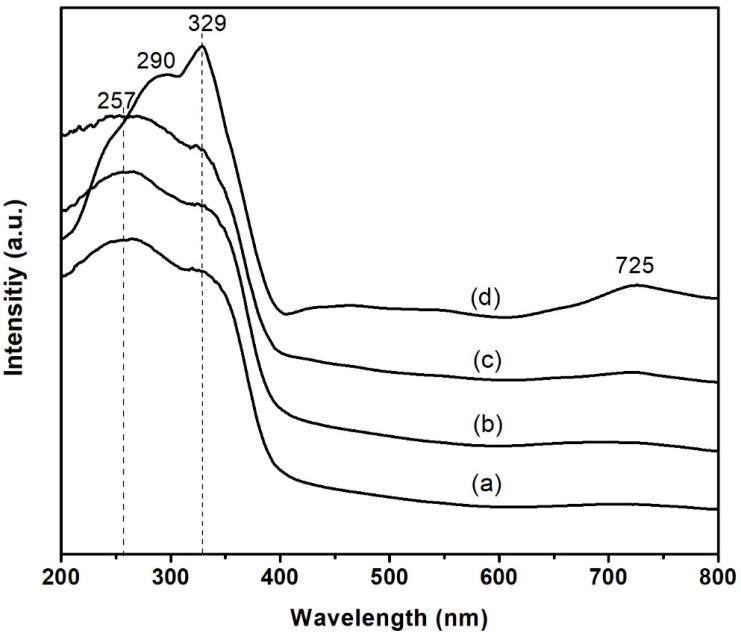
DRS UV-Vis spectra of (**a**) CeO_2_, (**b**) 9NiO/CeO_2_, (**c**) 27NiO/CeO_2_, and (**d**) 45NiO/CeO_2_ where the broad band centered at 725 nm shows the absorption band due to ^3^A_2g_(F) → ^3^T_1g_ (F) electronic transition of Ni^2+^ ions in octahedral coordination, viz. indicative of the covalent character in Ni-O_support_ metal-ligand bonds.

The electronic states of NiO and CeO_2_ in the *x*NiO/CeO_2_ samples were studied using DRS UV-Vis spectroscopy. A peak at 257 nm is observed in all Ni^2+^-containing samples, indicating the occurrence of charge transfer (CT) transitions involving Ni^2+^ ions (Figure 6b–d). These Ni^2+^ ions from NiO exist in octahedral form in the crystal lattice and are surrounded by six oxygen ions [39]. On the other hand, a peak at the nearly same position is also observed for the pristine CeO_2_, which corresponds to the CT transition from Ce^4+^ (4*f*) to O^2−^ (2*p*) (Figure 6a) [40]. CeO_2_ has a complicated electronic structure because it contains both Ce^3+^ and Ce^4+^ ions, both of which can undergo CT transitions with the oxygen ions in the crystal lattice [41,42]. Thus, this result suggests that there is a chemical interaction occurring between the NiO and CeO_2_ support. A peak of maximum absorption resulting from the charge transfer of O^2−^ → Ce^4+^ is identified at 290 nm, and a wide absorption edge of CeO_2_ is found at 329 nm [43,44]. Moreover, the peak intensity increases with the quantity of NiO impregnated, and this signal can be associated with the Ni-O charge transfer transition [45]. In addition, the 9NiO/CeO_2_, 27NiO/CeO_2_, and 45NiO/CeO_2_ exhibit a new and broad signal centered at 725 nm, which is attributed to the electronic transition of octahedrally coordinated Ni^2+^ ions from the ^3^A_2g_(F) → the ^3^T_1g_(F) state [46,47]. This band is stronger for 45NiO/CeO_2_, followed by 27NiO/CeO_2_ and 9NiO/CeO_2_. Thus, this band indicates a substantial covalent character in Ni-O_support_ metal-ligand bonds after calcination [46].

**Figure 7:**
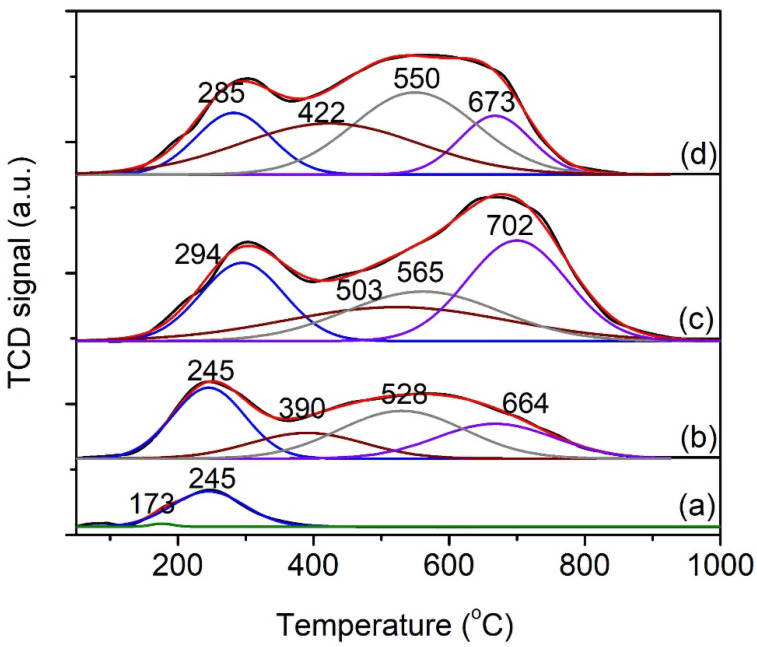
NH_3_-TPD plot of (**a**) CeO_2_, (**b**) 9NiO/CeO_2_, (**c**) 27NiO/CeO_2_, and (**d**) 45NiO/CeO_2_ with deconvolution.

**Figure 8:**
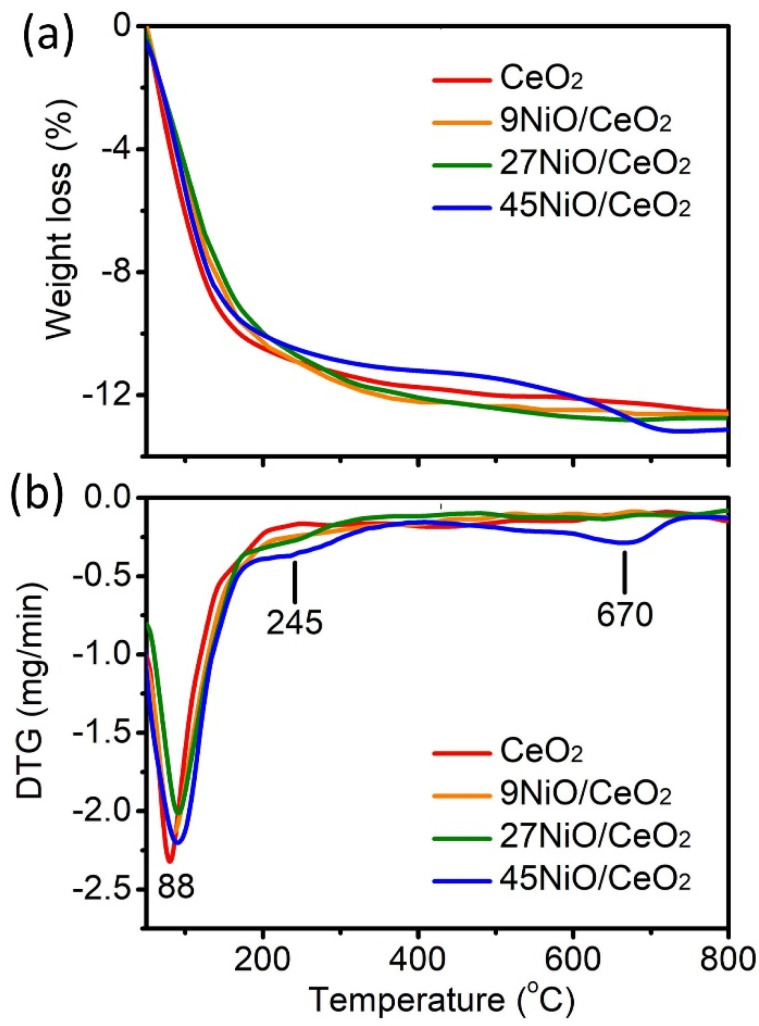
(**a**) TGA and (**b**) DTG curves of CeO_2_, 9NiO/CeO_2_, 27NiO/CeO_2_, and 45NiO/CeO_2_.

The acidity of *x*NiO/CeO_2_ solids was studied with the NH_3_-TPD, and the results are presented in Figure 7. It is shown that all the samples exhibit similar desorption tendency except for the bare CeO_2_, where the results can be divided into three desorption regions corresponding to the specific basic sites, namely mild acid sites (<300 °C), mild to strong acid sites (300–500 °C), and strong acid sites (>500 °C). The data reveal that 27NiO/CeO_2_ displays a larger desorption peak area and stronger peak intensity than pure CeO_2_, 9NiO/CeO_2_, and 45NiO/CeO_2_ catalysts, indicating the presence of larger quantity of acidic sites on the surface. The acidity data are also tabulated as in Table 1. From the data, it can be observed that the total acidity of the samples increases with increasing the NiO content. For pristine CeO_2_, it has acid sites with mild strength (129 μmol/g), which increases to 271 μmol/g and 320 μmol/g after the NiO content is increased to 9% and 27%, respectively, before slightly dropping to 235 μmol/g for 45NiO/CeO_2_. Similarly, the acidities with mild-to-strong and strong strengths also increase with increasing the NiO content. It should be noted that the 45NiO/CeO_2_ has a trace amount of non-reacted NiCl_2_, as indicated by TG/DTG and XRD (Figure 1 and Figure 8).

Thermogravimetric analysis/differential thermal analysis (TGA/DTG) was performed under air atmosphere to evaluate the thermal stability and heat resistance of the *x*NiO/CeO_2_ solids (Figure 8). The pristine CeO_2_ demonstrates two distinct stages of weight loss. The initial stage (<200 °C) is attributed to the removal of physiosorbed water (10.4%) and the second step of weight loss from 200 until 500 °C (1.7%) can be attributed to chemisorption of water and condensation of surface Ce–OH groups into a Ce–O–Ce group [48]. Upon functionalizing with NiO, an additional step of weight loss at 245 °C is observed by 9NiO/CeO_2_, 27NiO/CeO_2_, and 45NiO/CeO_2_, and the degree of weight loss increases with the amount of NiO incorporated. Meanwhile, 45NiO/CeO_2_ shows an additional weight loss centered at 670 °C (1.4%) due to the decomposition of non-reacted NiCl_2_ and Ni(OH)_2_. The data are in line with the XRD data indicating the presence of little NiCl_2_ and Ni(OH)_2_ phases in the 45NiO/CeO_2_ solid.

Hence, the desorption of NH_3_ at around 670 °C by 45NiO/CeO_2_ is also accompanied by the decomposition of non-reacted NiCl_2_ and Ni(OH)_2_, leading to the detection of the quantity of mild-to-strong and strong acid sites larger than the actual values. Among the three *x*NiO/CeO_2_ catalysts, 27NiO/CeO_2_ has the highest number of acidity (1564 μmol/g) with the highest acid strength (373 μmol/g). Thus, the NH_3_-TPD data clearly show that the incorporation of NiO on CeO_2_ (particularly 27NiO/CeO_2_) enhances the number of acid sites, which would promote enhanced catalytic activity for acetylation of glycerol reaction.

### 3.2. Catalytic Study

#### 3.2.1. Catalytic Comparative Study of xNiO/CeO_2_ Catalysts

Acetylation of glycerol was catalyzed by CeO_2_, 9NiO/CeO_2_, 27NiO/CeO_2_, and 45NiO/CeO_2_ under instant conductive heating (150 °C and 170 °C for 15 min) using an ethanoic acid:glycerol molar ratio of 10:1 and a catalyst loading of 0.04 g (Table 2). The reaction products obtained are monoacetin (MAT), diacetin (DAT), and triacetin (TAT). Pure CeO_2_ support is inactive in this reaction, which only converts 20.0% of glycerol into 19.3% of MAT and 72.7% of DAT at 150 °C (Entry 1). Upon elevating the temperature to 170 °C, the glycerol conversion exhibited an increase to 31.1%. The product selectivities were observed to be 17.2%, 72.6%, and 0% for MAT, DAT, and TAT, respectively. However, the reaction conversion increases upon impregnating NiO nanoparticles on the CeO_2_, with the catalytic activity enhanced In the following sequence: CeO_2_ < 9NiO/CeO_2_ < 45NiO/CeO_2_ < 27NiO/CeO_2_. Among these solids, the 27NiO/CeO_2_ shows the best results, with the highest glycerol conversion of 84.3% and 95.3% at 150 °C and 170 °C, respectively, and selectivity to DAT (66.0% and 60.4%) (Entry 3). The high activity of 27NiO/CeO_2_ can be explained by its large amount and good dispersal of active NiO nanoparticles (with strong acidity) on the support that are highly accessible for the adsorption and activation of reactant molecules. In contrast, the 45NiO/CeO_2_ catalyst exhibits poorer catalytic activity than the 27NiO/CeO_2_ solid due to its lower surface area with fewer octahedral NiO species incorporated, as confirmed by the nitrogen adsorption isotherm and DRS UV-Vis spectroscopy results. Additionally, pore blockage could also happen due to the excessive amount of Ni species present on the CeO_2_ surface, which inhibits molecular diffusion to the active sites, thereby causing poor catalytic performance [49]. Thus, the 27NiO/CeO_2_ catalyst was chosen to further study the acetylation of glycerol involving reaction temperature, heating time, catalyst loading, glycerol to ethanoic acid ratio, and catalyst reusability.

#### 3.2.2. Effect of Reaction Temperature and Duration

**Figure 9:**
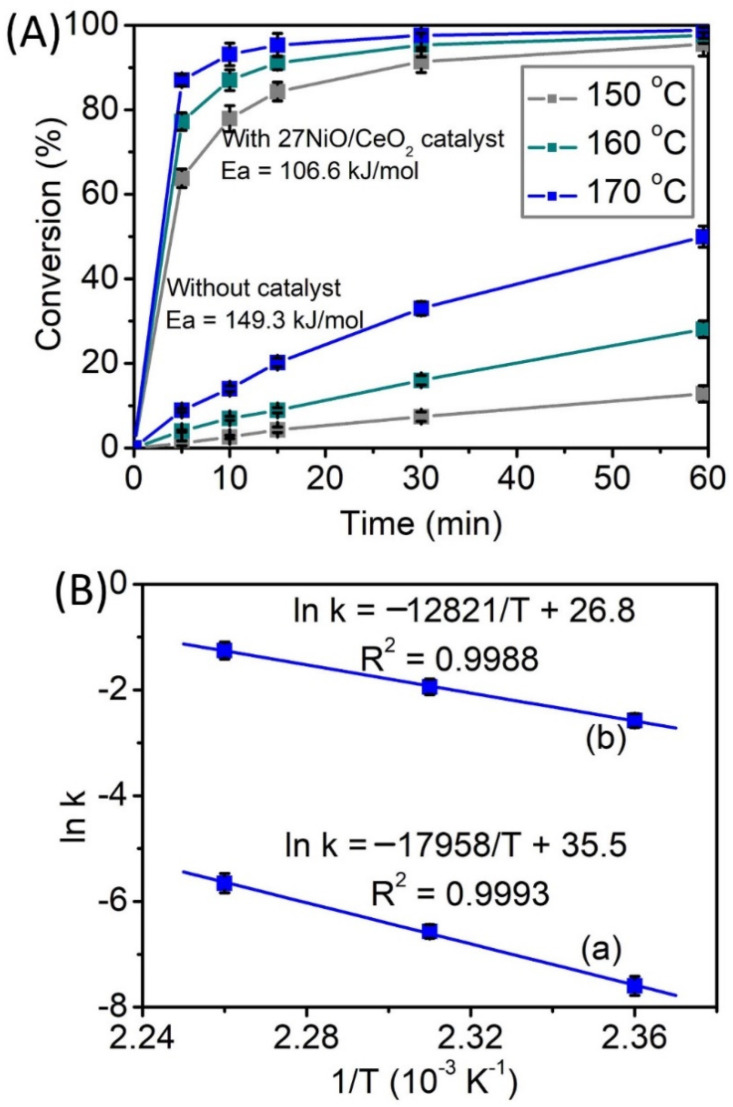
(**A**) Effects of reaction temperature (150 °C, 160 °C, and 170 °C)) and time (0–60 min) on the acetylation of glycerol with ethanoic acid in the presence and absence of 27NiO/CeO_2_ catalyst. (**B**) Arrhenius linear plots plotted using second-order rate constants obtained at 150 °C, 160 °C, and 170 °C.

The acetylation reaction over the 27NiO/CeO_2_ catalyst was investigated from 0 to 60 min at 150 to 170 °C. As seen in Figure 9A, the reaction temperature is the dominant factor affecting the glycerol conversion. For instance, at 150 °C, the conversion is 63.8% within 5 min and the conversion increases to 95.5% after 60 min. However, when the temperature increases from 150 °C to 170 °C, a substantial increase in the conversion from 63.8% to 87.0% is observed after 5 min. The reaction time extends to 60 min; the conversion (95.5%) at 150 °C increases to 97.6% and 98.8% for 160 °C and 170 °C, respectively. The observed phenomenon may be attributed to the increased frequency of effective collisions between glycerol and ethanoic acid at elevated temperatures, resulting in a high reaction rate [50]. At 170 °C, the conversion increases to 97.6% and becomes nearly stable after 30 min. The activation energies (E_a_) of the reaction catalyzed with 27NiO/CeO_2_ and without a catalyst are calculated using the Arrhenius equation to further understand the catalytic effect on the reaction kinetics where second-order rate constants are applied (Figure 9B). The E_a_ of the non-catalyzed reaction is 149.3 kJ mol^−1^ and the value decreases tremendously to 106.6 kJ mol^−1^ after catalyzing with 27NiO/CeO_2_. Thus, this indicates that the acetylation reaction has been activated, where 27NiO/CeO_2_ provides another reaction route of lower activation energy. Based on the study, the optimum reaction temperature and time are 170 °C and 30 min, respectively.

#### 3.2.3. Effect of the Amount of Catalyst

**Figure 10:**
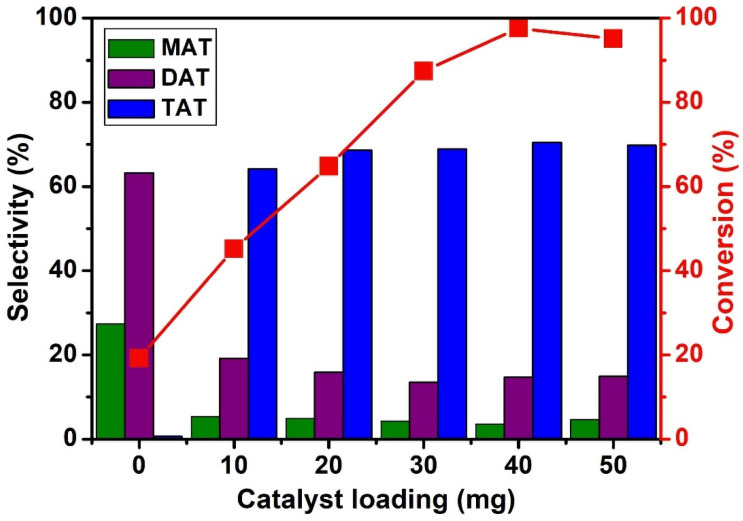
Conversion of glycerol and selectivity of MAT, DAT, and TAT catalyzed using different 27NiO/CeO_2_ catalyst loadings. Reaction conditions: glycerol:ethanoic acid molar ratio = 1:10, temperature = 170 °C, time = 30 min, solvent-free.

A catalyst is needed in a transformation reaction to ensure cost effectiveness of a process [51]. Hence, the 27NiO/CeO_2_ catalyst loading ranging from 0 to 0.05 g was studied with other variables remaining unchanged (Figure 10). Only 17.8% of glycerol is converted into 27.4% of MAT and 63.2% of DAT after 30 min at 170 °C. Upon introducing 0.01 g of catalyst, the conversion reaches 45.2%. The conversion is again enhanced to 64.8% and 87.4% when the amount of catalyst is increased to 0.02 g and 0.03 g, respectively. The optimum catalyst loading is 0.04 g, with 97.6% conversion is achieved at the same reaction time. However, upon increasing the catalyst loading to 0.05 g, the glycerol conversion slightly decreases (95.1%) due to inefficient stirring and mixing resulting from excessive catalyst loading [52].

An increase in conversion is anticipated with catalyst loading as a result of the availability of more accessible active sites. However, the products’ selectivity exhibits the opposite trend as the catalyst loading is altered. For instance, the selectivity towards DAT is predominant without a catalyst since the glycerol conversion is low, and the formation of TAT increases with the amount of catalyst. The 27NiO/CeO_2_ catalyst at a dosage of 0.04 g showed the best performance (97.6% conversion, 70.5% selective to TAT). To our knowledge, this result is the best catalytic performance thus far [22,23,53,54]. For the subsequent catalytic reaction experiments, 0.04 g of 27NiO/CeO_2_ was used.

#### 3.2.4. Effect of Glycerol:Ethanoic Acid Molar Ratio

**Figure 11:**
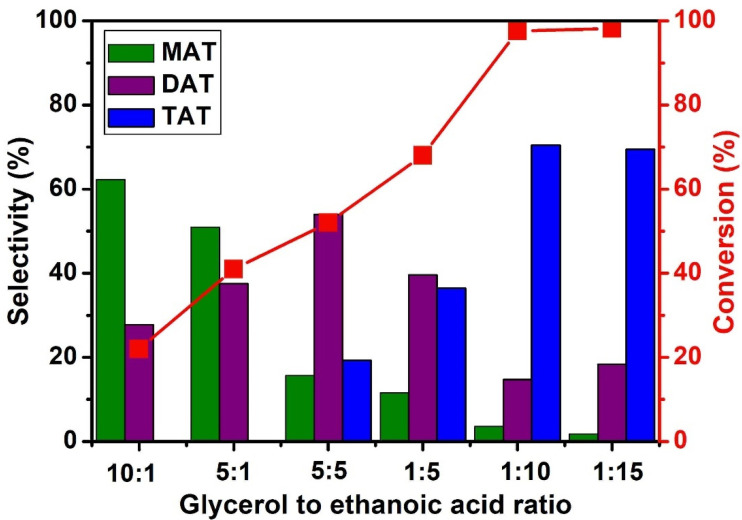
Effect of glycerol:ethanoic acid molar ratio on acetylation of glycerol catalyzed by 27NiO/CeO_2_. Reaction conditions: temperature = 170 °C; time = 30 min; catalyst amount = 0.04 g, solvent-free.

Acetylation reaction performance can be greatly affected by the quantity of reactants used since they govern the reaction chemical kinetics [55]. In order to obtain the highest conversion and TAT selectivity, the amount of glycerol and ethanoic acid (in molar ratio) was varied, with other reaction parameters remaining identical. The conversion is low (22.0%) and no TAT is obtained (MAT: 62.3%, DAT: 27.8%) when the molar ratio of glycerol to ethanoic acid is 10:1 (Figure 11). When the glycerol:ethanoic acid ratio is reduced to 5:1, a less viscous reaction mixture is formed. As seen, the conversion increases one-fold (41.0%), and an increased selectivity of MAT and DAT of 51.0% and 37.4%, was recorded, respectively. This clearly indicates that a low glycerol concentration is beneficial for the reaction conversion as it eases reactant mixing and stirring, thereby improving product selectivity.

The acetylation is also studied by increasing the concentration of ethanoic acid. As equimolar reactants (5:5) were applied, the conversion was further enhanced to 52.0%, shifting the product distribution towards higher substituted product (15.7% MAT, 54.0% DAT, and 19.3% TAT). Thus, the results show that ethanoic acid activation is essential for enabling high catalytic activity [56]. In order to further prove this statement, the glycerol:ethanoic acid ratio was again increased to 1:10, where a significant reaction conversion enhancement was shown (up to 97.6%) and the TAT selectivity was doubled (70.5%) thanks to the high availability of activated ethanoic acid in the reaction medium. Beyond this ratio (1:15), the reaction performance was almost intact. Therefore, the optimum glycerol:ethanoic acid ratio is 1:10.

#### 3.2.5. Catalyst Recyclability Study

**Figure 12:**
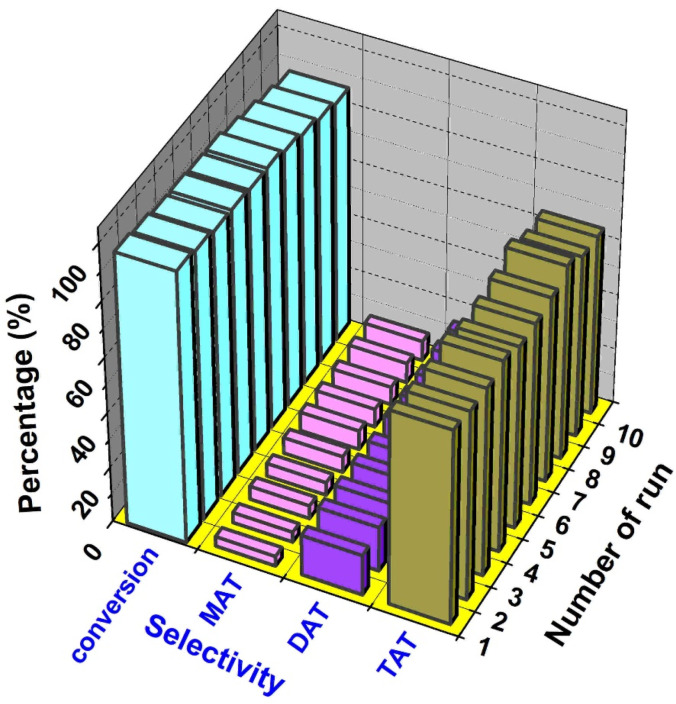
Catalyst reusability test for 27NiO/CeO_2_ solid in the acetylation of glycerol with ethanoic acid. Reaction conditions: glycerol:ethanoic acid molar ratio = 1:10; reaction temperature = 170 °C; reaction time = 30 min; catalyst loading = 0.04 g.

The deactivation of catalyst active sites is a crucial issue for a heterogeneous catalysis system [5]. Therefore, a reusability test was conducted on 27NiO/CeO_2_ to evaluate its stability and industrial feasibility. Figure 12 shows the glycerol conversion and products’ selectivity for ten consecutive runs. The glycerol conversion decreases from 97.6% to 86.0% after ten cycles of reuse. Hence, the reduction in the conversion could have resulted from the deactivation of active sites and partial leaching of active metal species from the surface of the catalyst support [57]. In addition, the product distribution shifted towards lower substituted products after multiple reaction runs. For instance, the MAT and DAT selectivities increased from 3.6% to 7.3% and 14.7% to 14.9%, respectively, after the 10th run. However, a slight reduction in the TAT selectivity was recorded from 70.5% to 65.2%, which indicates that the 27NiO/CeO_2_ catalyst is somewhat stable up to ten runs.

### 3.3. Thermodynamics and Kinetics Properties of Reaction

**Figure 13:**
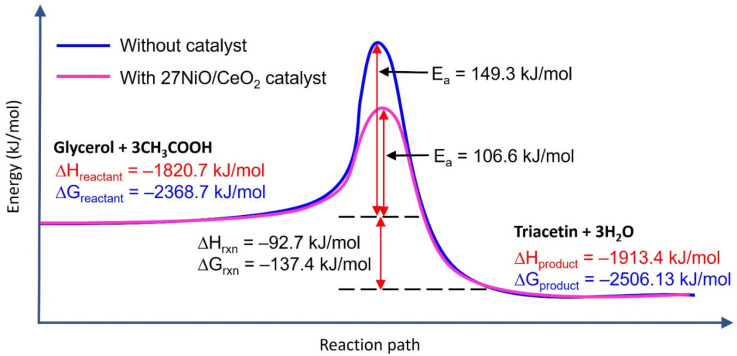
Energy profiles of acetylation of glycerol into triacetin with and without the 27NiO/CeO_2_ catalyst at 170 °C.

The production of ethyl levulinate in the presence of the 27NiO/CeO_2_ catalyst affects the kinetics and thermodynamic properties. Hence, in order to explain this phenomenon, the thermodynamic quantities (∆H_rxn_, ∆S_rxn_ and ∆G_rxn_) of acetylation of glycerol at 170 °C were calculated using the data provided in Refs. [58,59,60] and the following equations:

(3) $$∆H_{reaction}=∆H_{298K}^{o}+\int_{298}^{443}∆C_{P}dT$$

(4) $$∆S_{reaction}=∆S_{298K}^{o}+\int_{298}^{443}\frac{∆C_{P}}{T}dT$$

(5) $${∆G}_{reaction}={∆H}_{reaction}-T{∆S}_{reaction}$$

where enthalpy and entropy of vaporization (∆H_vap_) values of ethanoic acid and water are also included. The energy profile of the reaction is summarized as in Figure 13. The calculation reveals that the acetylation of glycerol with ethanoic acid reaction is an exothermic reaction with ∆H_rxn_ = −92.7 kJ/mol. Meanwhile, the ∆G_rxn_ is calculated to be −137.4 kJ/mol, revealing that the acetylation reaction is very spontaneous at 170 °C. Although this reaction is thermodynamically favorable, it occurs at a very slow speed in the absence of a catalyst. This can be proven when mild conversion (50.2%) is observed at 170 °C after 60 min in the absence of a catalyst (Figure 9A). Thus, the Gibbs free energy cannot address this particular concern (how fast does the reaction achieve high conversion). The chemical kinetics are hence the tools needed to address it.

The reaction kinetics (or rate constants) are very reliable on the temperature. When high temperature is applied, the reaction takes place at a faster rate but the activation energy is still unchanged [59]. Hence, glycerol in mild conversion (~50%) is recorded at 170 °C after 60 min (Figure 9A). The presence of the 27NiO/CeO_2_ catalyst is important in the acetylation of glycerol as it provides a different reaction route by allowing the diffusion, adsorption, and close proximity between reactant molecules on the active sites of the 27NiO/CeO_2_ catalyst before they are converted into TAT. As a result, this new reaction pathway lowers the activation energy from 149.3 kJ/mol to 106.6 kJ/mol and enhances the reaction kinetics.

### 3.4. Possible Mechanism of the Acetylation Reaction of Glycerol

**Figure 14:**
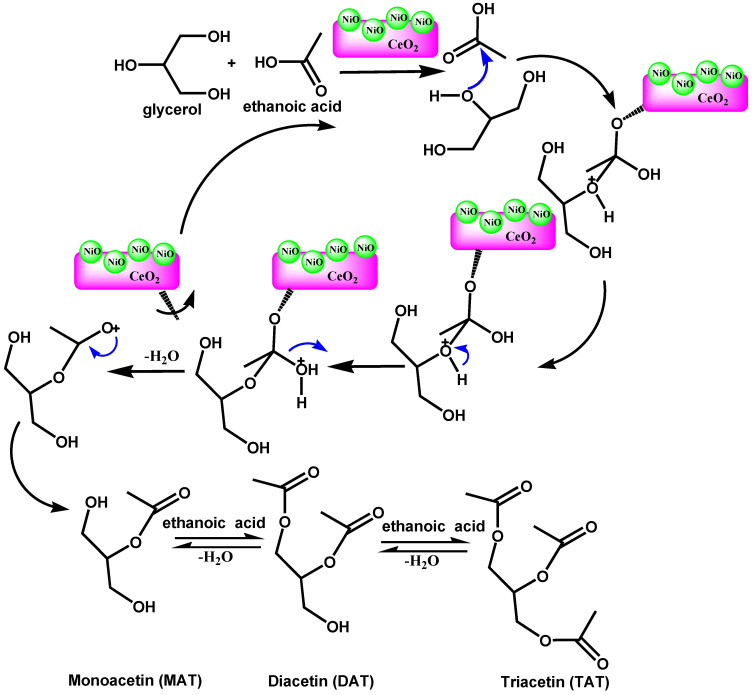
Plausible reaction mechanism for the acetylation of glycerol to yield monoacetin (MAT), diacetin (DAT), and triacetin (TAT) over the 27NiO/CeO_2_ catalyst.

The proposed mechanism is based on the current results and previous research [61]. The most plausible pathway for the acetylation of glycerol with ethanoic acid involves the activation of the carbonyl group of ethanoic acid by NiO in a 27NiO/CeO_2_ catalyst, which increases the electrophilicity of the carbonyl carbon (Figure 14). CeO_2_, on the other hand, is a redox catalyst and is capable of providing oxygen vacancies to aid the catalytic reaction process. The oxygen in glycerol attacks the carbonyl carbon, resulting in the formation of an intermediate species. The proton is transferred from the intermediate to the second hydroxyl group of glycerol, leading to the formation of an activated complex and a water molecule. Monoacetin (MAT) is formed subsequently. The aforementioned mechanism is augmented by the interaction of MAT with ethanoic acid, which forms diacetin (DAT) and triacetin (TAT). Besides increasing the glycerol conversion, the addition of both NiO and CeO_2_ also accelerates and increases the selectivity of the glycerol acetylation reaction. The mechanism combines acid–base and redox chemistry, which is facilitated by the task-specific properties of the incorporated NiO and CeO_2_ support.

## 4. Conclusions

In summary, NiO incorporated onto CeO_2_-supported solids (*x*NiO/CeO_2_) was prepared, and these solids were used as highly selective catalysts for glycerol acetylation. The XRD, SEM, and TEM data confirm that the NiO nanoparticles are well dispersed on the CeO_2_ support, and the chemical interactions between the NiO nanoparticles and CeO_2_ support are proven by the DRS UV-Vis spectroscopy. Furthermore, enhanced conversion of glycerol to MAT, DAT, and TAT products with ethanoic acid was successfully performed with all the prepared catalysts (CeO_2_, 9NiO/CeO_2_, 27NiO/CeO_2_, and 45NiO/CeO_2_). 27NiO/CeO_2_ appears to be the best catalyst in catalyzing the glycerol acetylation, with very high efficiency (97.6% glycerol conversion; 70.5% selectivity towards TAT) under optimum conditions, where the formation of by-products can be suppressed thanks to its strong acidity and high accessible surface area. Thus, this study demonstrates that 27NiO/CeO_2_ is very promising for catalyzing glycerol within a short reaction time (<30 min), thanks to the instant heating mode of the Monowave 50 reactor. Furthermore, the catalyst is reusable (up to 10 times) with minimal effect on the reaction performance. We thus believe that 27NiO/CeO_2_ will be a very promising heterogeneous catalyst and has high potential for synthesizing renewable biofuels in the petrochemical industry.

## Figures and Tables

**Table 1:** Physicochemical properties of *x*NiO/CeO_2_ samples.

Samples	NiO Content (%)	BET Surface Area (m^2^/g)	Micropore Surface Area (nm)	Average Pore Diameter (nm)	Total Pore Volume (cm^3^/g)	Particle Size (nm)	TPD-NH_3_ Acidity (μmol/g)			
							Mild	Mild-to-Strong	Strong	Total
CeO_2_	0	69	12	9.86	0.15	125 ± 23	129	0	0	129
9NiO/CeO_2_	8.10	71	7	8.95	0.13	124 ± 33	271	473	217	961
27NiO/CeO_2_	28.10	31	4	7.51	0.07	120 ± 33	320	871	373	1564
45NiO/CeO_2_	44.02	1	0	5.93	0.002	149 ± 30	235	1123 *	154 *	1512 *

* The values also include the decomposition of non-reacted NiCl_2_ and Ni(OH)_2_.

**Table 2:** Acetylation of glycerol over *x*NiO/CeO_2_ catalysts.

Entry	Sample	Conversion (%) at 150 °C	Products Selectivity (%) at 150 °C			Conversion (%) at 170 °C	Products Selectivity (%) at 170 °C		
			MAT	DAT	TAT		MAT	DAT	TAT
1	CeO_2_	20.0	19.3	72.7	0.0	31.1	17.2	72.6	0.0
2	9NiO/CeO_2_	77.5	22.0	66.3	2.2	88.0	19.6	55.3	13.6
3	27NiO/CeO_2_	84.3	18.2	66.0	4.3	95.3	15.8	60.4	14.9
4	45NiO/CeO_2_	76.0	20.3	66.7	2.9	87.2	17.6	57.0	14.8

Reaction conditions: ethanoic acid:glycerol molar ratio = 10:1; catalyst amount = 0.04 g; time = 15 min; solventless.

## Data Availability

The data presented in this study are available on request from the corresponding author.
